# Alcohol Septal Ablation or Septal Myectomy? An Updated Systematic Review and Meta-Analysis of Septal Reduction Therapy for Hypertrophic Obstructive Cardiomyopathy

**DOI:** 10.3389/fcvm.2022.900469

**Published:** 2022-05-25

**Authors:** Xifeng Zheng, Bin Yang, Haosheng Hui, Bing Lu, Yinhui Feng

**Affiliations:** ^1^Department of Geriatrics, Affiliated Hospital of Guangdong Medical University, Zhanjiang, China; ^2^Department of Cardiology, Affiliated Hospital of Guangdong Medical University, Zhanjiang, China; ^3^Department of Nuclear Medicine, Affiliated Hospital of Guangdong Medical University, Zhanjiang, China

**Keywords:** hypertrophic obstructive cardiomyopathy, alcohol septal ablation, septal myectomy, meta-analysis, systematic review

## Abstract

**Objective:**

To evaluate the safety and effectiveness of alcohol septal ablation (ASA) and septal myectomy (SM) for the treatment of hypertrophic obstructive cardiomyopathy.

**Methods:**

We searched the PubMed, MEDLINE, EMBASE, and CBM databases for observational research articles related to ASA and SM published from the establishment of the databases to November 2021. All ultimate selected articles were highly related to our target. The Newcastle-Ottawa Scale was used to evaluate the literature quality. A fixed or random effect model was performed in the meta-analysis depending on the heterogeneity of the included studies. The Mantel-Haenszelt test with relative risk ratio (RR) and 95% confidence interval (CI) was used to measure the effect indicator of binary data, while the inverse variance method with weighted mean difference (WMD) and 95% CI was used to measure the effect indicator of continuous data.

**Results:**

A totally of 3,647 cases (1,555 cases treated with ASA and 2,092 cases treated with SM) were included. The results of the systematic review indicated no statistically significant difference in postoperative all-cause mortality (RR = 0.82; 95% CI: 0.65–1.04; *P* = 0.10) between patients treated with ASA and SM, but both the reduction in the postoperative left ventricular outflow tract pressure gradient (WMD = 9.35 mmHg, 95% CI: 5.38–13.31, *P* < 0.00001) and the post-operative improvement on cardiac function, assessed by the grade of New York Heart Association (NYHA), compared to pre-operative measurements (WMD = 0.13; 95% CI: 0.00–0.26; *P* < 0.04) in the ASA group were slightly inferior to those in the SM group. In addition, both the risk of pacemaker implantation (RR = 2.83, 95% CI: 2.06–3.88; *P* < 0.00001) and the risk of reoperation (RR = 11.23, 95% CI: 6.21–20.31; *P* < 0.00001) are recorded at a higher level after ASA procedure.

**Conclusion:**

Both ASA and SM have a high degree of safety, but the reduction in the postoperative left ventricular outflow tract pressure gradient and the improvement on cardiac function are slightly inferior to SM. In addition, both the risk of pacemaker implantation and the risk of reoperation are recorded at a higher level after ASA procedure. The operative plan should be chosen through multidisciplinary discussions in combination with the wishes of the patients and the actual clinical situation.

## Introduction

Hypertrophic cardiomyopathy (HCM) is a heritable heart disease defined as spontaneous genetic mutations probably, isolated, and progressive myocardial hypertrophy. Gene mutations that encode proteins of the cardiomyocytic contractile apparatus (thick and thin myofilament contractile of the cardiac sarcomere) are major causes of HCM. The disease has obvious clinical heterogeneity, ranging from asymptomatic patients with a positive genotype to patients with typical symptoms of angina pectoris or heart failure and even to the extreme state with sudden cardiac death as the first symptom. It is also the main cause of sudden cardiac death in young people and athletes, according to epidemiological investigations (1, 2). HCM can be divided into two phenotypes, obstructive and non-obstructive, based on the hemodynamic characteristics. Left ventricular outflow tract obstruction is a specific risk factor for sudden cardiac death. Septal reduction therapy is the main non-pharmaceutical therapy for medically refractory hypertrophic obstructive cardiomyopathy (i.e., symptomatic with a poor drug treatment effect and a left ventricular outflow tract pressure gradient ≥50 mm Hg at rest or excitation) (3). Surgical treatment with septal myectomy (SM), considered the “gold standard” for the treatment of severe hypertrophic obstructive cardiomyopathy, can reduce the left ventricular outflow tract pressure gradient, release the degree of outflow tract obstruction, and improve the cardiac function and prognosis (4). With the continuous development of cardiac interventional technology and the emergence of new therapeutic instruments, alcohol septal ablation (ASA) has accumulated considerable supporting clinical evidence because of it causes less trauma with a rapid recovery, especially for elderly patients or patients with a variety of chronic diseases (5).

However, a comparison of ASA and SM with respect to the ability to reduce the left ventricular outflow tract pressure gradient and improve cardiac function, the risk of perioperative complications, short -and long-term postoperative mortality, and other issues requires further analysis. Although Liebregts et al. performed systematic retrospective analyses on the long-term outcomes of SM and ASA in 2015 (6), however, their study did not present direct comparisons of both strategies. Moreover, the data range of the extant research is outdated, and many newly published research data were not included in the analysis. Therefore, this study aimed to present head-to-head comparisons the efficiency and safety of ASA and SM by integrating the latest data to provide insights for clinical treatment.

## Materials and Methods

### Literature Retrieval Strategy

The authors collected data from the literature published until November 2021 from several sources, namely, the China Biomedical Literature (CBM), PubMed, MEDLINE, and EMBASE databases. The keywords used were “hypertrophic cardiomyopathy,” “alcohol septal ablation,” and “septal myectomy.” We adopted a search strategy that combined the medical subject headings and text words. Meanwhile, other strategies, such as the manual retrieval of literature, were employed to supplement potentially relevant literature.

### The Criteria of Literature Inclusion and Exclusion

The inclusion criteria were as follows: (1) Participants: Patients with hypertrophic cardiomyopathy; (2) Study type: cohort studies; (3) Intervention measures: alcohol septal ablation was considered as the experimental group, while septal myectomy was considered as the control group; and (4) Outcome indicators: all-cause mortality (number of deaths / total number of patients × 100%), reduction of postoperative left ventricular outflow tract pressure gradient, improvement on cardiac function, and the risk of permanent pacemaker implantation.

The exclusion criteria were as follows: (1) studies without controls; (2) case studies or special case reports; (3) incomplete or un-extractable data on outcome indicators; (4) reviews; and (5) animal experiments.

### Data Extraction and Literature Quality Evaluation

Two authors (Zheng and Feng) used Microsoft Excel to extract and organize the data of the included literature.

#### Data Extraction

We screened the literature in strict accordance with the inclusion and exclusion criteria. Publications were preliminarily excluded based on the title and abstract and then further screened based on the full text. The information extracted mainly included: (1) title, corresponding authors, and publication time; (2) methodological quality evaluation elements; (3) the gender, age, sample size, time, and specific intervention methods of patients in the treatment and control groups; and (4) outcome indicators.

#### Literature Quality Evaluation

The Newcastle-Ottawa Scale (7) was used to evaluate the literature quality and contained three sections with eight subsections: (1) Exposure: representativeness of the exposed cohort, selection of the non-exposed cohort, ascertainment of exposure, and the demonstration that the outcome of interest was not present at the start of the study; (2) Comparability: comparability of cohorts based on the design or analysis; and (3) Outcome: an assessment of outcome, whether the follow-up was long enough for outcomes to occur, and the adequacy of follow-up of the cohorts. A star-rating system was adopted, and the total possible score of a cohort study was 13.

### Statistical Method

The authors employed ReviewManager 5.3 software to conduct a systematic review analysis. The Mantel-Haenszelt test with relative risk ratio (RR) and 95% confidence interval (CI) was used to measure the effect indicator of binary data, while the inverse variance method with weighted mean difference (WMD) and 95% CI was used to measure the effect indicator of continuous data. A fixed or random effect model was performed in the meta-analysis depending on the heterogeneity of the included studies. The heterogeneity was determined by the *P* value obtained from Cochran's Q test and I^2^. For *P* value > 0.1 and I^2^ <50%, the heterogeneity among the included studies was considered not significant, and the fixed effect model was applied for further analysis. Otherwise, the heterogeneity among the studies was considered significant, and the publications were individually eliminated (leave-one-out method) for a sensitivity analysis and to compare the changes in heterogeneity before and after elimination to determine the source of heterogeneity. When the source of heterogeneity could not be determined or I^2^ was >50%, a random effects model was used. In addition, the Egger test (8) conducted by Stata 14 software and funnel chart were used to evaluate the publication bias of the included articles.

## Results

### Literature Retrieval and Quality Evaluation

**Figure 1:**
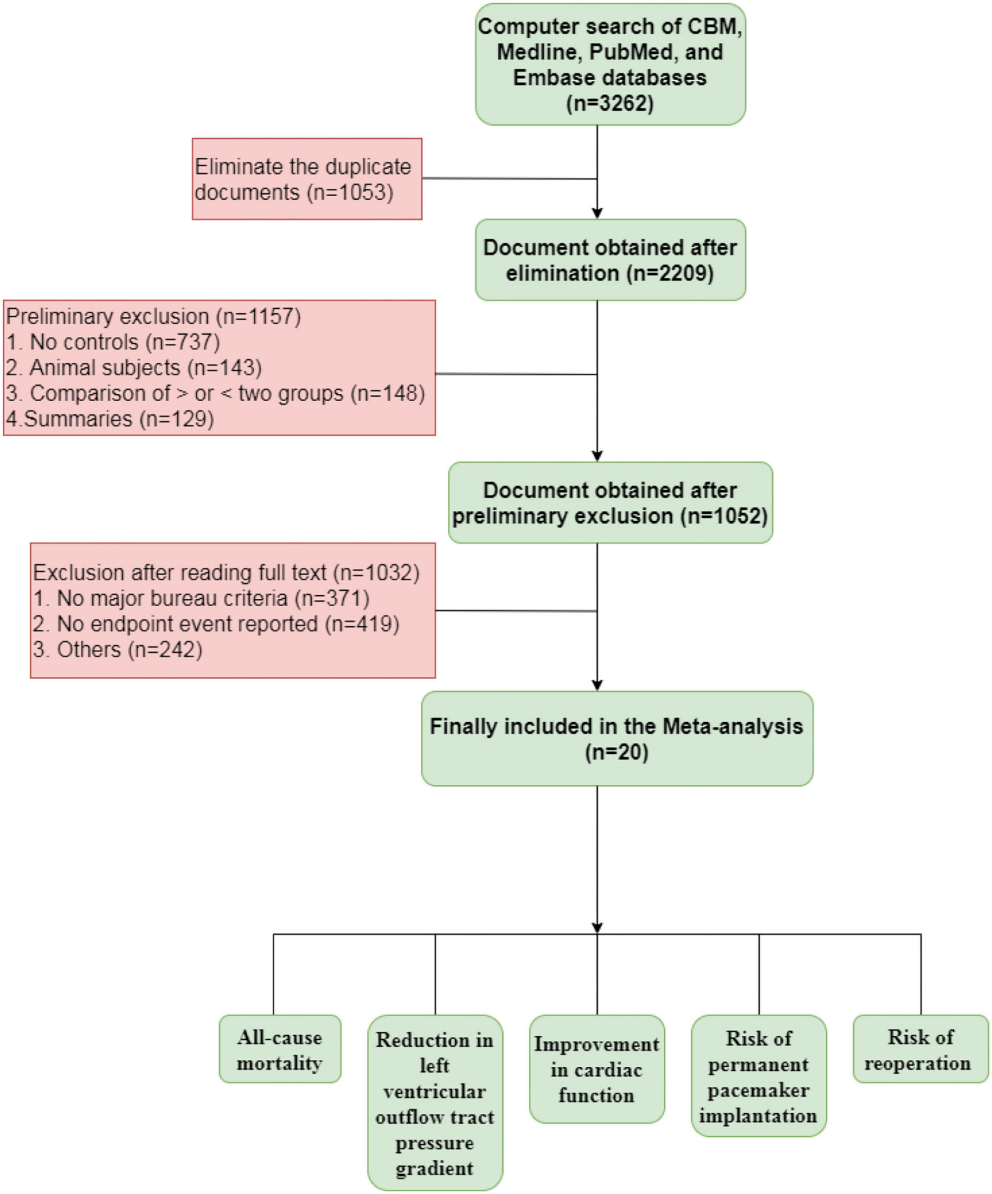
The flowchart of the study.

As shown in Figure 1, 3,262 relevant studies were preliminarily retrieved. Duplicate publications and irrelevant literature, based on their titles and abstracts, were excluded. Finally, 20 studies were included. Next, we evaluated the quality of the included studies, which were all cohort studies. The blinding method was not mentioned in the included studies. The 20 studies included in the meta-analysis are shown in Table 1.

**Table 1:** The scores of the 20 studies included in the meta-analysis assess by Newcastle-Ottawa Scale.

**References**	**Representativeness of the exposed cohort**	**Selection of the non-exposed cohort**	**Ascertainment of exposure**	**Demonstration that outcome of interest was not present at start of study**	**Comparability**	**Assessment of outcome**	**Was follow-up long enough for outcomes to occur**	**Adequacy of follow up of cohorts**	**Score**
Afanasyev et al. (9)	*	*	*	*	**	*	*		8
Hoedemakers et al. (10)	*	*	*	*	*	*	*	*	8
Kimmelstiel et al. (11)	*	*	*	*	**	*	*		8
Nguyen et al. (12)	*	*	*	*	**	*	*		8
Cavigli et al. (13)	*	*	*	*	**	*	*	*	9
Guo et al. (14)	*	*	*	*	*	*	*		7
Yang et al. (15)	*	*	*	*		*	*		6
Sedehi et al. (16)	*	*	*	*	**	*	*	*	9
Samardhi et al. (17)	*	*	*	*	*	*	*	*	8
Steggerda et al. (18)	*	*	*	*	*	*	*		7
Vriesendorp et al. (19)	*	*	*	*	**	*	*	*	9
Knyshov et al. (20)	*	*	*	*	*	*	*	*	8
Soraijja et al. (21)	*	*	*	*	**	*	*		8
Nagueh et al. (22)	*	*	*	*	**	*	*		8
Vural et al. (23)	*	*	*	*	*	*	*	*	8
Ralph-Edwards et al. (24)	*	*	*	*	*	*	*	*	8
Van der lee et al. (25)	*	*	*	*	*	*	*	*	8
Teng-yong et al. (26)	*	*		*	*	*	*	*	7
Firoozi et al. (27)	*	*	*	*	**	*	*	*	9
Xin et al. (28)	*	*		*	*	*	*		6

* *means one point and*

** *means two points under the newcastle-ottawa scale*.

### All-Cause Mortality in Patients With Hypertrophic Cardiomyopathy

**Figure 2:**
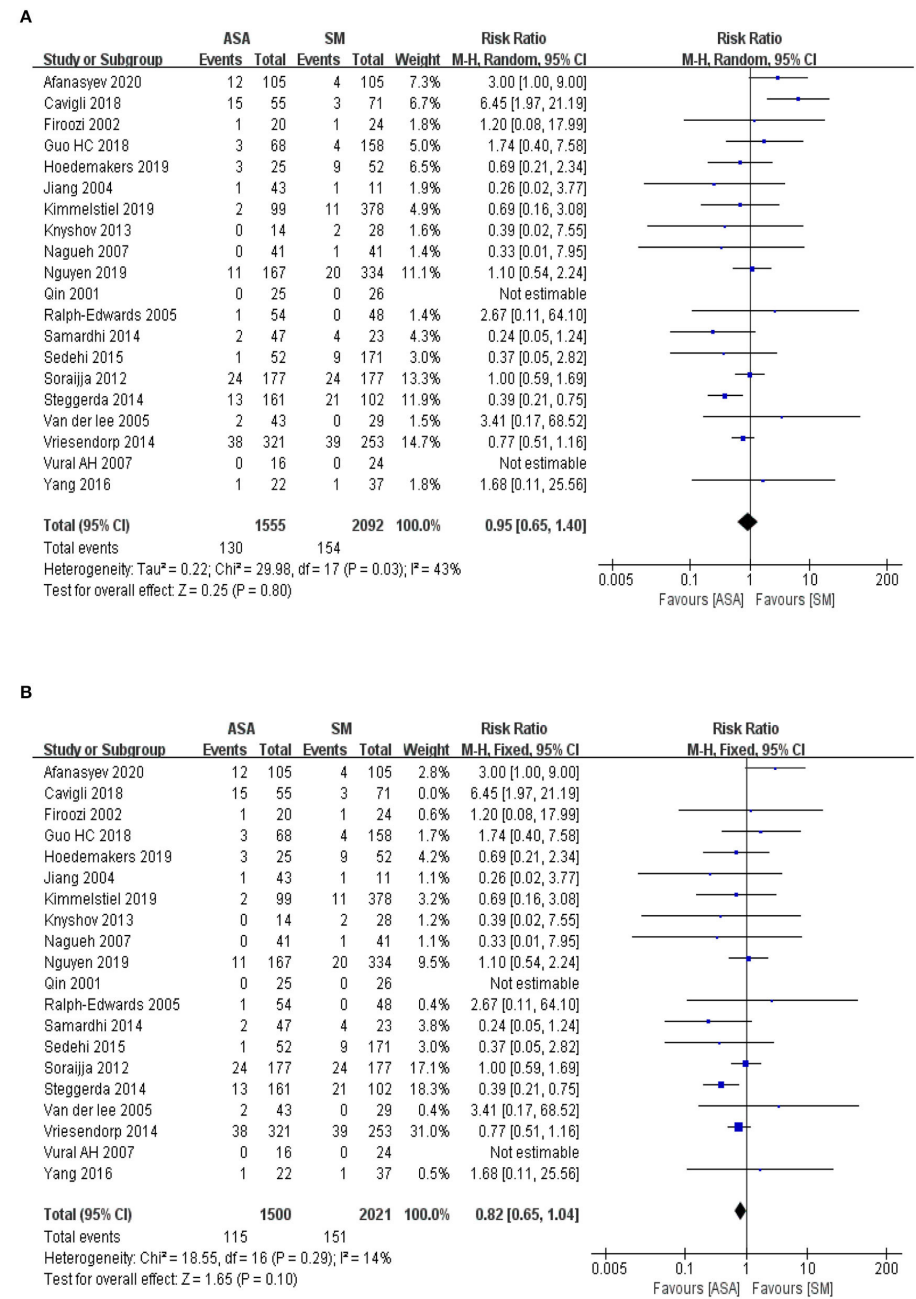
**(A)** The forest map for the meta-analysis of all-cause mortality. M-H, Mantel-Haenszelt test; CI, confidence interval; Random, random effect model. **(B)** The adjusted forest map for the meta-analysis of all-cause mortality. M-H, Mantel-Haenszelt test; CI, confidence interval; Fixed, fixed effect model.

A total of 20 included articles provided statistics on the outcome index of all-cause mortality in patients with hypertrophic cardiomyopathy, including 3,647 total cases (ASA group, 1,555 cases; SM group, 2,092 cases). The meta-analysis used the relative risk ratio (RR) as the effect indicator, and Cochran's Q test was conducted on the heterogeneity of all included articles. The results of heterogeneity (Figure 2A, I^2^ = 43%, P = 0.03) indicated statistically significant heterogeneity among the studies. The sensitivity analysis was conducted using the “leave-one-out method”; this proved that the heterogeneity mainly origin from Cavigli's 2018 study. After excluding this study, the I^2^ score decreased from 43 to 14% and the *P* value of Cochran's Q-test increased to 0.29. In this case, we employed the Mantel-Haenszel test with a fixed effect model for the meta-analysis and the results (Figure 2B, RR = 0.82; 95% CI: 0.65–1.04; *P* = 0.10) indicate that although the all-cause mortality in the ASA group (7.67%) was slightly higher than that in the SM group (7.47%), the difference between the two groups was not statistically significant (*P* > 0.05).

### Reduction in Left Ventricular Outflow Tract Pressure Gradient

**Figure 3:**
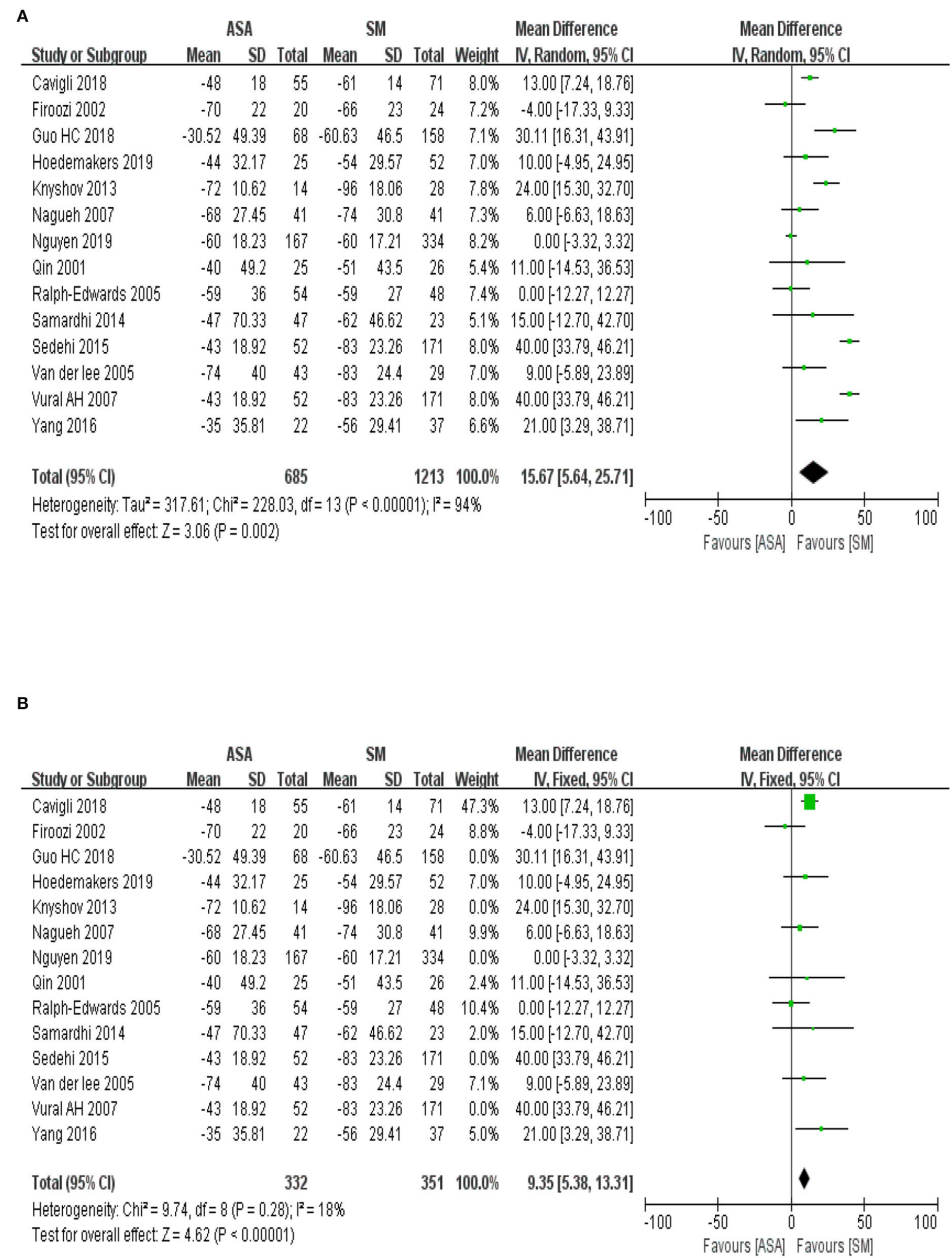
**(A)** The forest map for the meta-analysis of the reduction in left ventricular outflow tract pressure gradient. IV, inverse variance; CI, confidence interval; Random, random effect mod. **(B)** The adjusted forest map for the meta-analysis of the reduction in left ventricular outflow tract pressure gradient. IV, inverse variance; CI, confidence interval; Fixed, fixed effect model.

Fourteen articles were included in the present study. Statistics were performed on the outcome index of the left ventricular outflow tract pressure gradient in patients with hypertrophic cardiomyopathy, including 1,898 total cases (ASA group, 685 cases; SM group, 1,213). In the meta-analysis, WMD was used as the effect indicator, and a Cochran's Q-test was conducted on the heterogeneity. The results of heterogeneity (Figure 3A, I^2^ = 94%, *P* < 0.00001) indicated statistically significant heterogeneity among the studies. The sensitivity analysis was conducted using the “leave-one-out” method and proved that the heterogeneity mainly originated from studies by (12, 14, 16, 20, 23). After excluding these studies, the I^2^ score decreased from 94 to 18% and *P* value of Cochran's Q-test increased to 0.28. Therefore, the inverse variance method with the fixed effects model was used for the analysis. The results of the meta-analysis illustrated as Figure 3B (WMD = 9.35 mmHg, 95% CI: 5.38–13.31, *P* < 0.00001), which indicated that the reduction in the left ventricular outflow tract pressure gradient in the ASA group was lower than that in the SM group, and the difference between the two groups was statistically significant (*P* < 0.05).

### Improvement on Cardiac Function

**Figure 4:**
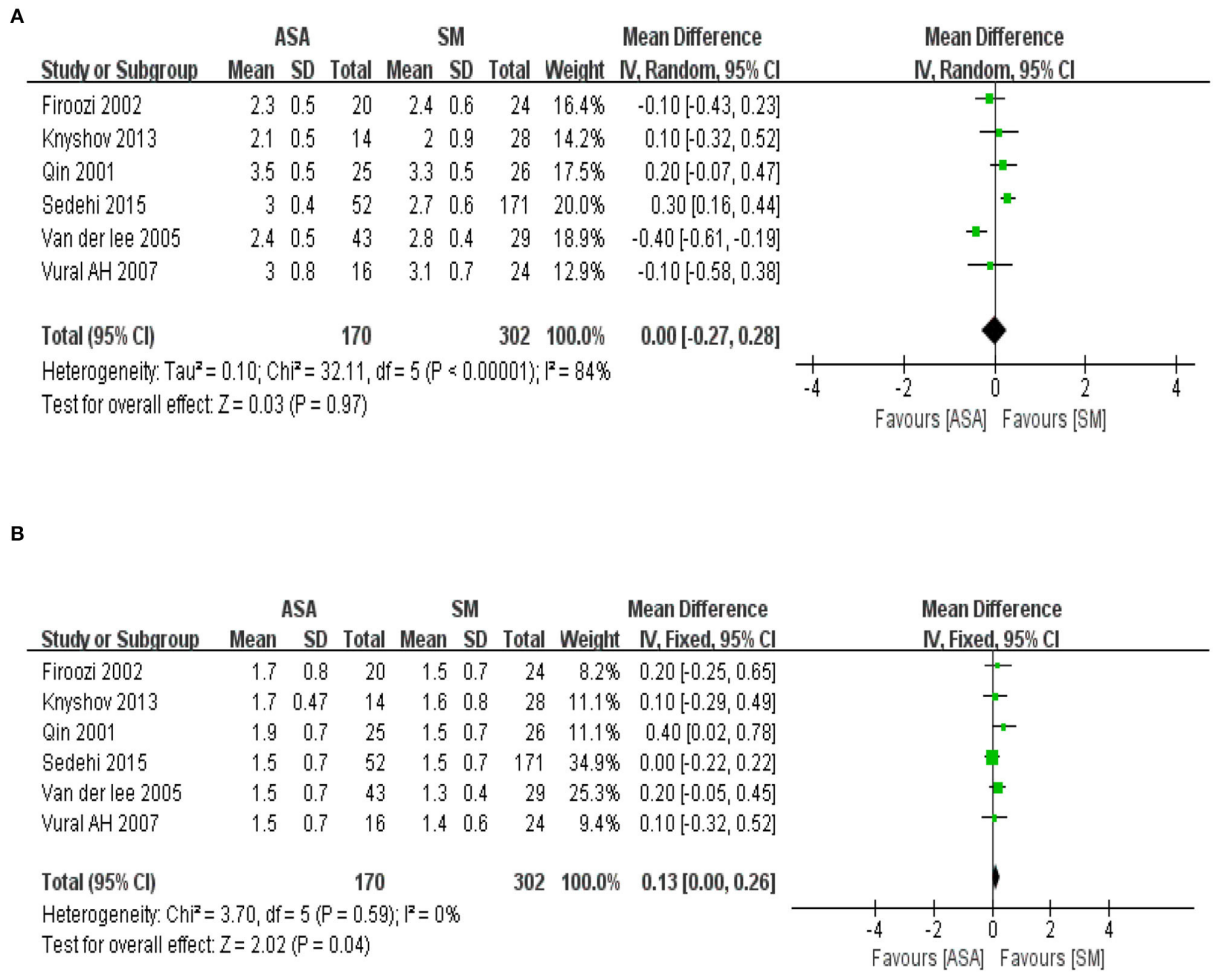
**(A)** The forest map of baseline information in cardiac function of the included studies. IV, inverse variance; CI, confidence interval; Random, random effect mod. **(B)** The forest map of improvement on cardiac function after the procedures. IV, inverse variance; CI, confidence interval; Fixed, fixed effect model.

A total of six included articles were analyzed to evaluate the improvement on cardiac function assessed by the grade of New York Heart Association (NYHA) before and after operation, including 472 total cases (ASA group with 170 cases; SM group with 302 cases). The baseline information of the cardiac function suggests no statistical difference exists between the ASA group and the SM group (Figure 4A, *P* = 0.97). To explore the improvement on the cardiac function after the procedures, the meta-analysis used WMD as an effect indicator and conducted a Cochran's Q-test on the heterogeneity. The results of heterogeneity (Figure 4B, I^2^ = 0%, *P* = 0.59) implied no heterogeneity among the studies. Therefore, the inverse variance method with a fixed effects model was used for further analysis. The results of the meta-analysis showed that the effect value (WMD = 0.13; 95% CI: 0.00–0.26; *P* = 0.04), indicating that improvements on cardiac function in the ASA group was slightly weaker than that in the SM group.

### Risk of Permanent Pacemaker Implantation

**Figure 5:**
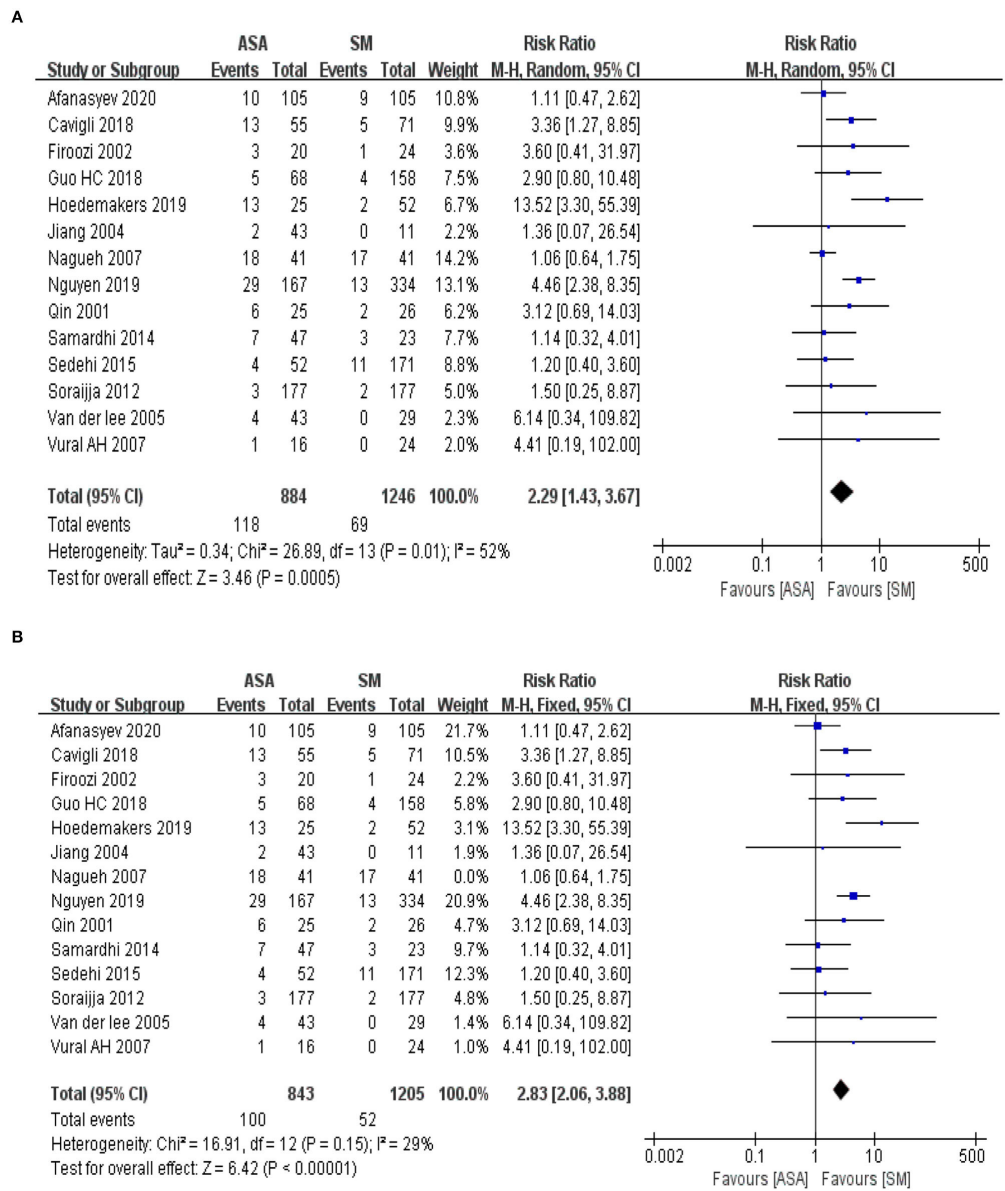
**(A)** The forest map for the Meta analyses of risk for permanent pacemaker implantation. M-H, Mantel-Haenszelt test; CI, confidence interval; Random, random effect model. **(B)** The adjust forest map for the Meta analyses of risk for permanent pacemaker implantation. M-H, Mantel-Haenszelt test; CI, confidence interval; Fixed, fixed effect model.

We assessed 14 included articles for the outcome index of pacemaker implantation rates in patients with hypertrophic cardiomyopathy, including total 2,130 cases (ASA group, 884 cases; SM group, 1,246 cases). The meta-analysis used RR as an effect indicator and conducted a Cochran's Q-test on heterogeneity. The heterogeneity results among the effect indicators (Figure 5A, I^2^ = 52%, *P* = 0.01) suggested that heterogeneity existed among the studies with statistical significance. In contrast, the sensitivity analysis by “leave-one-out” method indicated that I^2^ decreased from 52% to 29% and *P* value of Cochran's Q-test upgrade to 0.15 after excluding (22), implying that heterogeneity was mainly derived from this study. The fixed effect model with the Mantel-Haenszel method was used for further analysis. The effect value (Figure 5B, RR = 2.83, 95% CI: 2.06–3.88; *P* < 0.00001) indicated that the pacemaker implantation rate in the ASA group (11.86%) was significantly higher than that in the SM group (4.32%), and the difference between the two groups was statistically significant.

### Risk of Reoperation After ASA and SM Procedure

**Figure 6:**
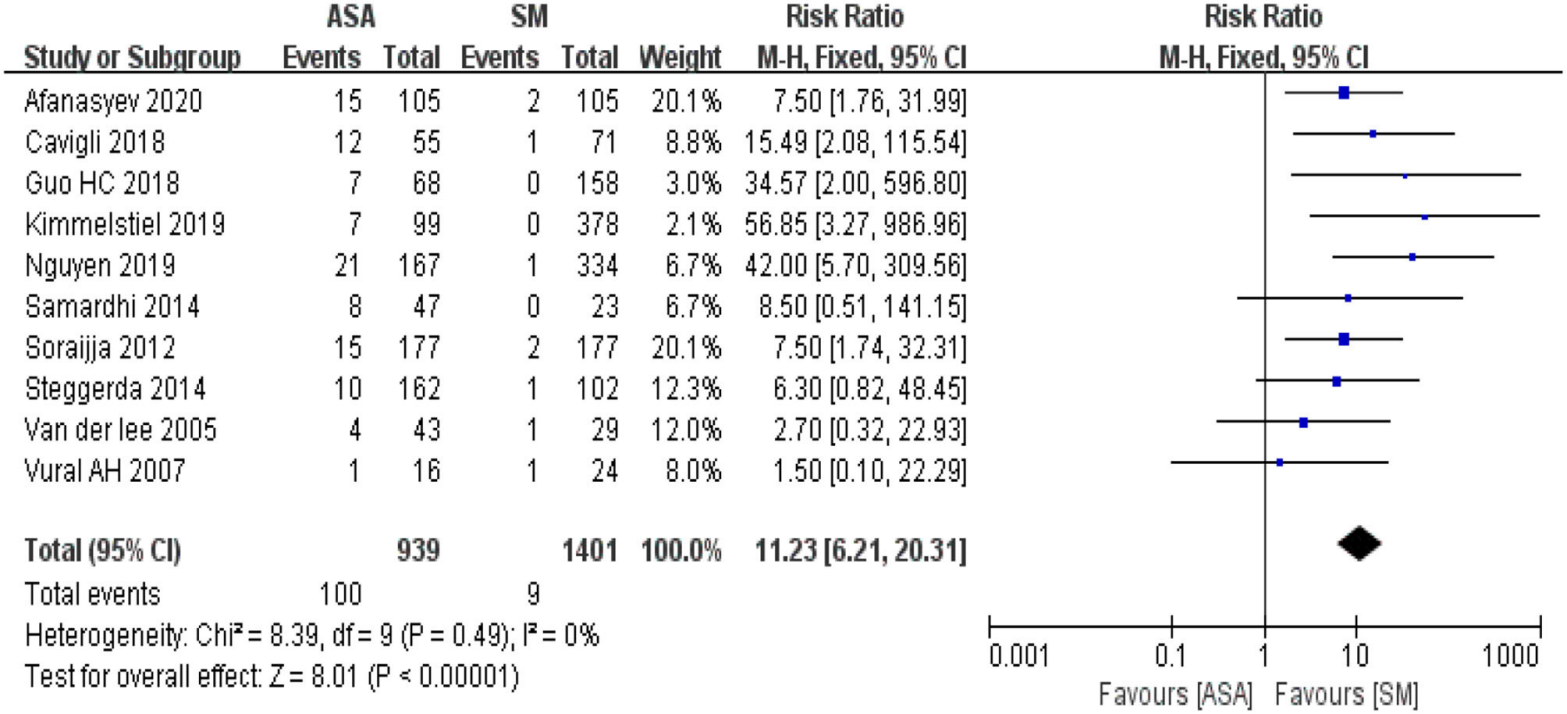
The forest map for the Meta analyses of risk of reoperation. M-H, Mantel-Haenszelt test; CI, confidence interval; Fixed, fixed effect model.

We identified 10 articles which assessed the risk of reoperation after ASA and SM in patients with hypertrophic cardiomyopathy, including a total of 2,340 cases (ASA group, 939 cases; SM group, 1,401 cases). The meta-analysis used RR as an effect indicator and conducted a Cochran's Q-test on heterogeneity. The heterogeneity results t indicators (Figure 6, I^2^ = 0%, *P* = 0.49) suggested that no heterogeneity existed among the studies. The fixed effect model with the Mantel-Haenszel method was used to assess the effect indicator. This resulted in RR = 11.23, 95% CI: 6.21–20.31; *P* < 0.00001, indicating that the risk of reoperation in the ASA group (10.65%) was significantly higher than that in the SM group (0.64%).

### Publication Bias

**Figure 7:**
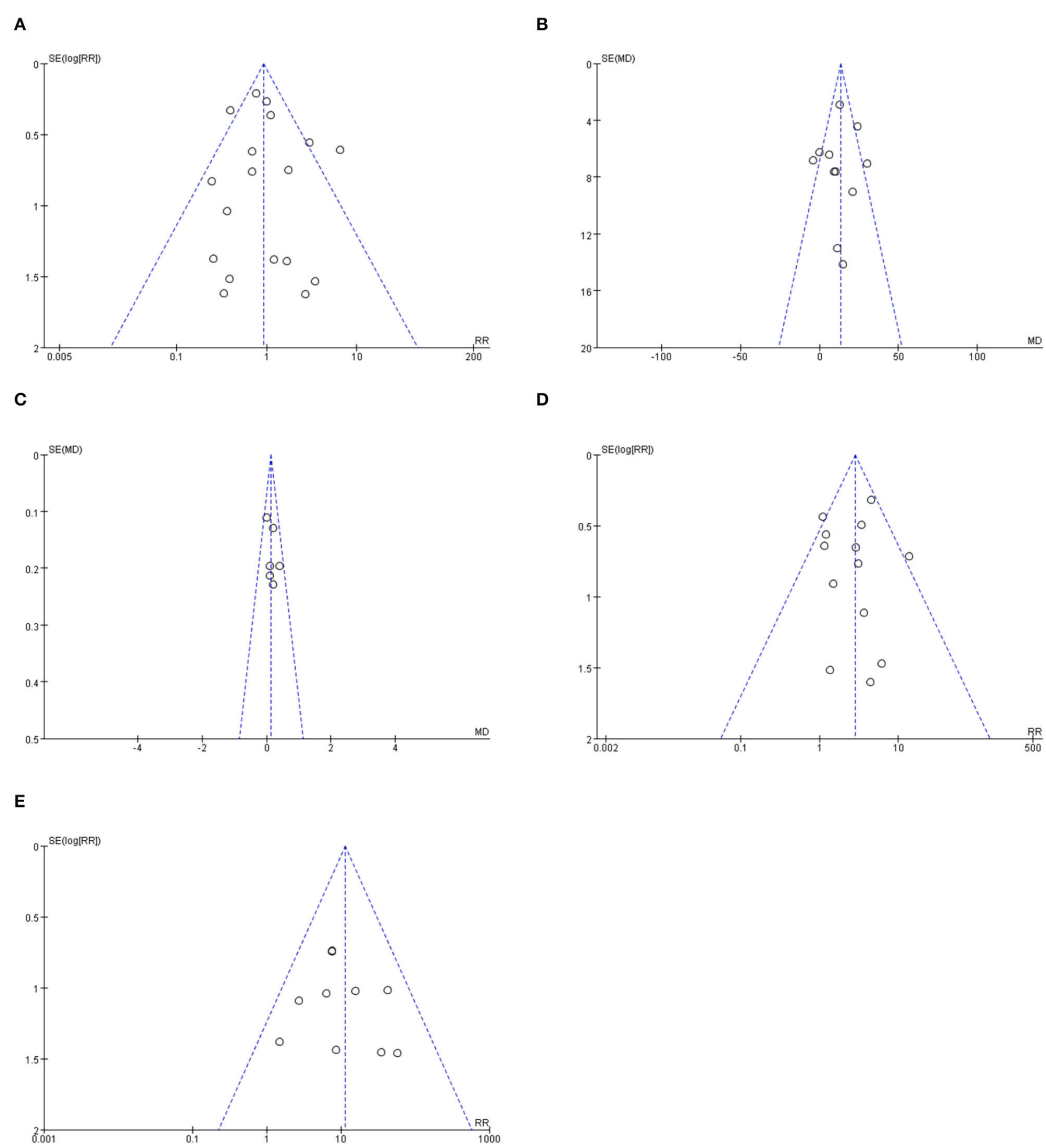
The funnel plot for the included studies of differential outcome indicators. **(A)** shows the funnel plot for publication bias of all-cause mortality related studies. **(B)** shows the funnel plot for publication bias of Reduction in left ventricular outflow tract pressure gradient. **(C)** shows the funnel plot for publication bias of improvement on cardiac function. **(D)** shows the funnel plot for publication bias of risk of permanent pacemaker implantation. **(E)** shows the funnel plot for publication bias of risk of reoperation.

The Egger test was used to explore the publication bias of the included studies, and visualization was performed using funnel plots. The included studies were evenly and symmetrically distributed on both sides of the axis, under the cover of the triangle in the funnel plots (Figure 7). The included studies were evenly and symmetrically distributed on both sides of the axis, under the cover of the triangle in the funnel plots. The combined results of the funnel plots and Egger test (*P* > 0.05) suggested that no publication bias existed.

## Discussion

SM therapy was first proposed by Morrow in 1961 (29) and was improved by Messmer in 1994 (30). It has a definite curative effect in clinical trials (31) and is considered the gold-standard treatment for medically refractory hypertrophic obstructive cardiomyopathy. However, rehabilitation after open-heart surgery remains a big challenge for the elderly and patients with chronic diseases. In 1995, Sigwart proposed a novel catheter-based technique (32) in which 1–4 ml absolute ethanol was injected into the proximal septal branch of the left anterior descending coronary artery to cause basal septal myocardial infarction and reduce the myocardial contractility of the septum. The ventricular septal myocardium subsequently became thinner and scarred, resulting in the widening of the left ventricular outflow tract, finally reducing or eliminating the left ventricular outflow tract pressure gradient. Compared with SM, ASA has the advantages of causing less trauma and a faster postoperative recovery; however, the long-term clinical results and prognosis remain to be further explored (33).

To explore the efficiency and perioperative risk of ventricular ASA, we conducted a systematic retrospective analysis of published clinical studies on SM and ASA. The results showed that there was no significant difference in all-cause mortality after ASA compared with that after surgical myectomy, which confirmed that ASA also has a high degree of safety, especially in experienced cardiac medical centers (34). Unfortunately, the indicators of the left ventricular outflow pressure gradient and improvement on cardiac function suggest that the relative effectiveness of ASA is slightly inferior to that of SM. In fact, not only did the size and distribution of septal vessels impact the effect of ASA but the formation of collateral circulation may limit the scope of myocardial infarction in the target area or aggravate myocardial ischemia in other parts of the left ventricle, resulting in a treatment effect that is hard to predict (35). Complete atrioventricular block is a common perioperative complication of ventricular ASA of the conduction system located in the posterior ventricular septal region. The risk of permanent pacemaker implantation in these patients is significantly higher than that in the SM group. High alcohol use during surgery is an important risk factor for a complete atrioventricular block (36).

The statistical results of clinical trials are a guide for clinical decision-making, rather than a dogma. Different surgical methods have specific target populations, are complementary to each other, and cover as many patients as possible. Doctors should fully explain the advantages and disadvantages of different surgical methods to patients and make reasonable decisions in combination with the wishes of patients and specific clinical conditions. The progress of medicine lies in continuous and reasonable innovations. The traditional view is that ASA is an alternative method for elderly patients who cannot tolerate the risks of surgery. At present, ASA has also achieved good results in clinical trials of young people aged 14–25 years, providing evidence for the expansion of its indications (37). With the accumulation of surgical experience and continuous improvement of surgical instruments, coupled with the expanding aging population, ventricular ASA may play a greater role in the future.

The main limitation of this study was that the included clinical studies were observational. Since HCM is a relatively rare disease, there are few patients who need septal reduction therapy, and its study is not suitable for randomized trials (38). There was some clinical heterogeneity in the included studies, which was unavoidable due to the comparison across different countries and populations.

## Conclusion

This updated meta-analysis included a total sample size of 3,647 hypertrophic obstructive cardiomyopathy patients (1,555 treated with ASA and 2,092 treated with SM) and presented head-to-head comparisons the efficiency and safety of ASA and SM. The results illustrated that there was no significant difference in all-cause mortality after ASA compared with patients after SM, but reduction in left ventricular outflow tract pressure gradient and the improvement on cardiac function were slightly inferior to those in the SM group, and has a higher risk of both pacemaker implantation and reoperation. The decision of operation plan should be decided by multidisciplinary discussion in combination with the wishes of patients and the actual situation.

## Data Availability Statement

The original contributions presented in the study are included in the article/supplementary material, further inquiries can be directed to the corresponding author.

## Author Contributions

XZ and YF was responsible for data extraction. XZ contributed in statistical analysis and original manuscript writing. YF was responsible for visualization. BY, HH, and BL were responsible for literature searching and preliminary screening. JC gave insightful advice for the revision. All authors contributed to the article and approved the submitted version.

## Funding

This study was funded by the Youth Scientific Research Program of Guangdong Medical University (GDMUM2020024).

## Conflict of Interest

The authors declare that the research was conducted in the absence of any commercial or financial relationships that could be construed as a potential conflict of interest.

## Publisher's Note

All claims expressed in this article are solely those of the authors and do not necessarily represent those of their affiliated organizations, or those of the publisher, the editors and the reviewers. Any product that may be evaluated in this article, or claim that may be made by its manufacturer, is not guaranteed or endorsed by the publisher.

## Abbreviations

HCM: Hypertrophic cardiomyopathy
ASA: Alcohol septal ablation
SM: septal myectomy
RR: relative risk ratio
CI: confidence interval
WMD: weighted mean difference
I^2^: I-square.
